# Inflammatory markers may predict post‐operative complications and recurrence in Crohn's disease patients undergoing gastrointestinal surgery

**DOI:** 10.1111/ans.17852

**Published:** 2022-06-22

**Authors:** Gil Mullin, Yaniv Zager, Roi Anteby, Harel Jacoby, Ilan Kent, Edward Ram, Ido Nachmany, Nir Horesh

**Affiliations:** ^1^ Department of Surgery and Transplantations B Chaim Sheba Medical Center Tel‐Hashomer Israel

**Keywords:** Crohn's disease, gastro‐intestinal surgery, outcomes, prognostic markers

## Abstract

**Background:**

Most Crohn's Disease (CD) patients will require surgical intervention over their lifetime, with considerably high rates of post‐operative complications. Risk stratification with reliable prognostic tools may facilitate clinical decision making in these patients. Blood cell interaction based inflammatory markers have proven useful in predicting patient outcomes in oncological and benign diseases. The aim of this study was to investigate their prognostic value in CD patients undergoing surgery.

**Methods:**

A retrospective single institution study of CD patients who underwent surgery between the years 2008 and 2019 was conducted. Data were collected from medical records and analysed for association of Platelet‐to‐Lymphocyte Ratio (PLR), Neutrophil‐to‐Lymphocyte Ratio (NLR), Lymphocyte‐to‐Monocyte Ratio (LMR) and the modified Systemic Inflammatory Score (mSIS) with post‐operative outcomes.

**Results:**

A total of 81 patients were included in the analysis. Half were females; mean age was 36 ± 15.54 years. Fifty seven percent (*n* = 46) were operated in expedited settings, with 23.5% developing post‐operative complications. In elective patients, higher pre‐operative NLR (*P* = 0.029) and PLR (*P* = 0.034) were associated with major post‐operative complications, higher NLR (*P* = 0.029) and PLR (*P* = 0.034) were associated with re‐operation and higher PLR correlated with Clavien‐Dindo score (*P* = 0.032). In patients operated in expedited operations, higher pre‐operative NLR (*P* = 0.021) and lower pre‐operative LMR (*P* = 0.018) were associated with thromboembolic events and higher mSIS was associated with major post‐operative complications (*P* = 0.032).

**Conclusions:**

Blood cell interaction based inflammatory markers confer an association with post‐operative complications in CD patients undergoing surgery. These indices may facilitate patient selection and optimization when considering the risks and benefits of surgical interventions.

## Graphical abstract

Blood cell interaction based inflammatory markers have proven useful in predicting patient outcomes in oncological and benign diseases. The aim of this study was to investigate their prognostic value in Crohn's Disease patients undergoing surgery.

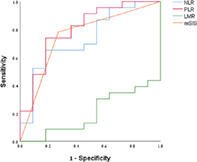

## Introduction

Crohn's disease (CD) is a chronic inflammatory condition characterized by a relapsing–remitting, progressive course. About one third of the patients diagnosed with non‐complicated disease, will develop a penetrating or stricturing disease within 5 years of diagnosis.^1^ Despite the advancements made with medical management of CD, and even in the era of biological‐agents based therapy—most patients will eventually require surgery.^2^, ^3^ The 10‐year risk of surgery has dropped over the past few decades, yet remains high and reaches up to 50% in some studies.^3^, ^4^ Surgery for CD is not curative, and many patients will require more than one surgical intervention during the course of their disease.^5^

Post‐operative complications occur more frequently in CD patients.^6^ This observation can probably be attributed to poor pre‐operative status caused by the underlying disease. Identification of markers that reflect the pre‐operative inflammatory and nutritional status can help stratify patients by risk level and may lead to better patient optimization prior to surgery. Improving pre‐operative patient optimization can help reduce surgery related morbidity and mortality.^7^

Several studies from the past few years have discussed the value of inflammatory markers based on blood cell interactions. Such markers are thought to represent the patient's inflammatory status, indicate the level of disease activity, and correlate with prothrombotic and malignant states. Platelet‐to‐Lymphocyte Ratio (PLR) is one emerging marker, that was shown to be valuable in both inflammatory^8^, ^9^ and malignant^10^, ^11^, ^12^, ^13^ conditions. Its value increases when it is interpreted in combination with other markers, such as the Neutrophil‐to‐Lymphocyte Ratio (NLR)—another predictive marker used both individually and in combination with others.^11^, ^12^, ^14^, ^15^, ^16^, ^17^ The Lymphocyte‐to‐Monocyte Ratio (LMR) is a predictive marker that was shown to be useful mainly in malignant diseases,^18^, ^19^, ^20^ and lately also in different bacterial infections^21^, ^22^ and other benign conditions such as osteoporosis.^23^ A prognostic score called the modified Systemic Inflammatory Score (mSIS) is based on LMR and serum albumin levels, that was also shown to be valuable in various malignant diseases^24^, ^25^, ^26^ and is yet to be examined in benign conditions.

Only few studies have previously examined the role of these inflammatory markers in CD patients. For example, recent studies have demonstrated a correlation between higher PLR and NLR values and CD diagnosis and activity,^27^, ^28^ while LMR and mSIS were not previously tested in CD. Only NLR was previously studied as a predictor of post‐operative complications in CD, with contradictory results.^29^, ^30^ PLR^31^ and LMR^32^ were studied as predictors for post‐operative complications in other conditions, but not in CD. No previous studies have assessed any of the markers as predictors for length of remission after surgery in CD patients.

In this study, we aimed to investigate the possible value of the blood cell interactions based inflammatory markers in predicting post‐operative complications and disease recurrence in a cohort of CD patients, who underwent gastrointestinal surgery in a large tertiary referral center.

## Material and methods

We performed a retrospective single institution study, including patients with formally diagnosed CD, who underwent surgery at a large tertiary referral center between the years 2008 and 2019. The study was approved by the local institutional ethics committee (SMC‐1443‐14).

Patient characteristics, medical background and pre‐operative, intraoperative, post‐operative and follow‐up data were collected and analysed. Severity of post‐operative complications was classified using the Clavien‐Dindo system.^33^ Major complications were defined as Clavien‐Dindo≥3. Surgery was defined expedited if performed within 1 week from acute admission and elective otherwise.^34^, ^35^

PLR, LMR, NLR and mSIS scores were calculated using pre‐operative data. Association analysis was performed for each prognostic score with post‐operative outcomes. A subgroup analysis was conducted according to the type of operation (expedited versus elective).

Statistical analysis was performed using SPSS (Version 25, IBM Corp, Armonk, NY) software. Associations between post‐operative complications and inflammatory markers were assessed using Area Under the Curve (AUC). Binary logistic regression was used to assess multivariate association between inflammatory markers and post‐operative complications. Bivariate correlations were calculated, using Pearson's coefficient for continuous variables and Spearman's coefficient for discrete variables, between inflammatory markers and the length of stay (LOS) and maximal Clavien‐Dindo score. Cox regression was used to evaluate univariate and multivariate association between inflammatory markers and recurrence. A *P*‐value of <0.05 was considered statistically significant.

## Results

### Patient characteristics

We reviewed the data of 121 patients who underwent ileocolic resection for CD between June 2009 and October 2018. Of them, 81 had sufficient and timely laboratory results data and were included in the final analysis. Forty‐one (50.6%) were female. The mean age was 36 ± 15.54 years. Forty‐six patients (56.8%) were operated in expedited settings. The mean disease duration at time of surgery was 10.03 ± 6.05 years. Other demographic and clinical data are presented in Table 1.

**Table 1:** Demographic and clinical characteristics of patients with Crohn's disease who underwent gastrointestinal surgery (*N* = 81)

Variable	*N*	Standard deviation	%
Mean age	36	15.54	
Gender			
Male	40		49.4
Female	41		50.6
Mean BMI (kg/m^2^)	21.51	4.02	
Smokers	11		13.8
Mean disease Duration (years)	10.03	6.05	
Pre‐operative corticosteroid use	25		32.1
Pre‐operative anti‐TNF‐α Use	15		19.2
Mean pre‐operative albumin (mg/dl)	3.23	0.65	
Operation urgency			
Expedited	46		56.8
Elective	35		43.2

Abbreviations: BMI, Body Mass Index; N, number.

### Post‐operative complications

While 19 patients (23.5%) presented with post‐operative complications, only 10 (12.3%) presented with major complications (Clavien‐Dindo score ≥ 3). Eight patients (9.9%) required re‐operation. Twenty‐four patients (29.6%) were readmitted within 30 days from the operation. Median LOS was 10 days (range 5–62 days). Other post‐operative outcomes are presented in Table 2.

**Table 2:** Post‐operative outcomes

Outcome	*N* (%)
Post‐operative complication rate	19 (23.5)
Major complications rate (Clavien‐Dindo ≥3)	10 (12.3)
Post‐operative abdominal abscess	11 (13.6)
Wound infection (SSI)	9 (11.1)
Paralytic ileus	3 (3.7)
Pneumonia	4 (4.9)
Thrombo‐embolic event	2 (2.5)
Cardio‐vascular event	4 (4.9)
Re‐operation rate	8 (9.9)
Re‐admission rate	24 (29.6)
Median LOS (days)	10 (range: 5–62)

Abbreviations: LOS, length of stay; SSI, surgical site infection.

#### Elective operations

**Fig. 1:**
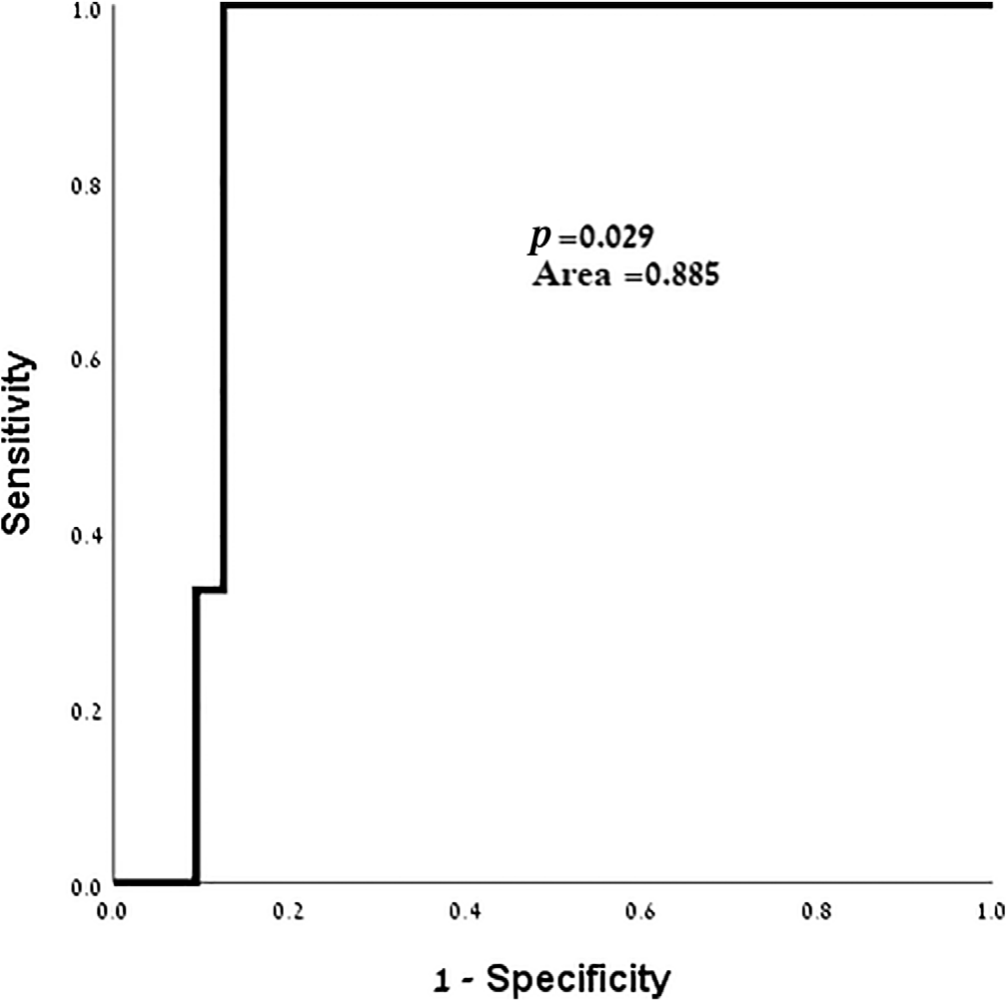
Area under the curve (AUC) analysis of NLR and major post‐operative complications (Clavien‐Dindo ≥3) in patients undergoing elective procedures. *P* = *P*‐value.

Area under the curve (AUC) analysis demonstrated that among patients who were operated in elective settings, the incidence of major post‐operative complications was associated with NLR (*P* = 0.029, *Area* = 0.885, Fig. 1) and with PLR (*P* = 0.034). Re‐operation was associated with NLR (*P* = 0.029) and PLR (*P* = 0.034). However, these associations were not found to be independent in multivariate analysis. Data is presented in Table 3. Clavien‐Dindo score correlated with PLR (*P* = 0.032), as presented in Table 4. No correlation was demonstrated between outcome measures in elective patients and LMR or mSIS.

**Table 3:** Area under the curve analysis of inflammatory markers and surgical outcomes in expedited and elective procedures (*P*‐value)

	NLR		PLR		LMR		mSIS	
	Expedited	Elective	Expedited	Elective	Expedited	Elective	Expedited	Elective
Post‐operative Complications	0.514	0.986	0.602	0.824	0.72	0.772	0.11	0.878
Major Post‐operative Complications (CD≥3)	0.819	0.029*	0.194	0.034*	0.216	0.377	0.032*	0.976
Surgical Wound Infection (SSI)	0.91	0.366	0.275	0.969	0.97	0.844	0.598	0.829
Paralytic Ileus	0.09	0.477	0.187	0.943	0.105	0.831	0.114	0.32
Pneumonia	0.236	0.076	0.451	0.102	0.067	0.776	0.451	0.594
Anastomotic Leak	‐	0.063	‐	0.152	‐	0.72	‐	0.197
Abscess	0.602	0.93	0.862	0.791	0.543	0.227	0.685	0.702
Thrombo‐embolic Event	0.021*	‐	0.957	‐	0.018*	‐	0.451	‐
Cardio‐vascular Event	0.09	0.175	0.187	0.099	0.105	0.195	0.114	0.216
Re‐operation	0.819	0.029*	0.251	0.034*	0.986	0.377	0.138	0.976
Re‐admission	0.991	0.365	0.806	0.706	0.202	0.792	0.412	0.308

Abbreviations: NLR, Neutrophil to Lymphocyte Ratio; PLR, Platelet to Lymphocyte Ratio; LMR, Lymphocyte to Monocyte Ratio; mSIS, Modified Systemic Inflammatory Score; CD, Clavien‐Dindo.

* *P* < 0.05.

**Table 4:** Inflammatory markers correlations with length of stay and Clavien‐Dindo score

		NLR		PLR		LMR		mSIS	
		Coefficient	PV	Coefficient	PV	Coefficient	PV	Coefficient	PV
Expedited operations	LOS	0.277	0.065	0.251	0.096	−0.116	0.447	0.089	0.56
	Clavien‐Dindo	0.06	0.69	−0.194	0.197	0.185	0.219	−0.234	0.117
Elective operations	LOS	0.315	0.074	0.341	0.052	−0.28	0.115	0.28	0.115
	Clavien‐Dindo	0.295	0.095	0.364	0.032 *	−0.152	0.385	0.106	0.549

Abbreviations: PV, *P*‐value.

* *P* < 0.05.

#### Expedited operations

Area under the curve (AUC) analysis demonstrated that among patients who were operated in expedited settings the incidence of thromboembolic events was associated with higher NLR (*P* = 0.021) and lower LMR (*P* = 0.018). The incidence of major post‐operative complications was associated with mSIS (*P* = 0.032). However, these associations were not found to be independent in multivariate analysis. Data is presented in Table 3. No correlation was demonstrated between outcome measures in patients who underwent expedited operations and PLR.

### Disease recurrence

**Fig. 2:**
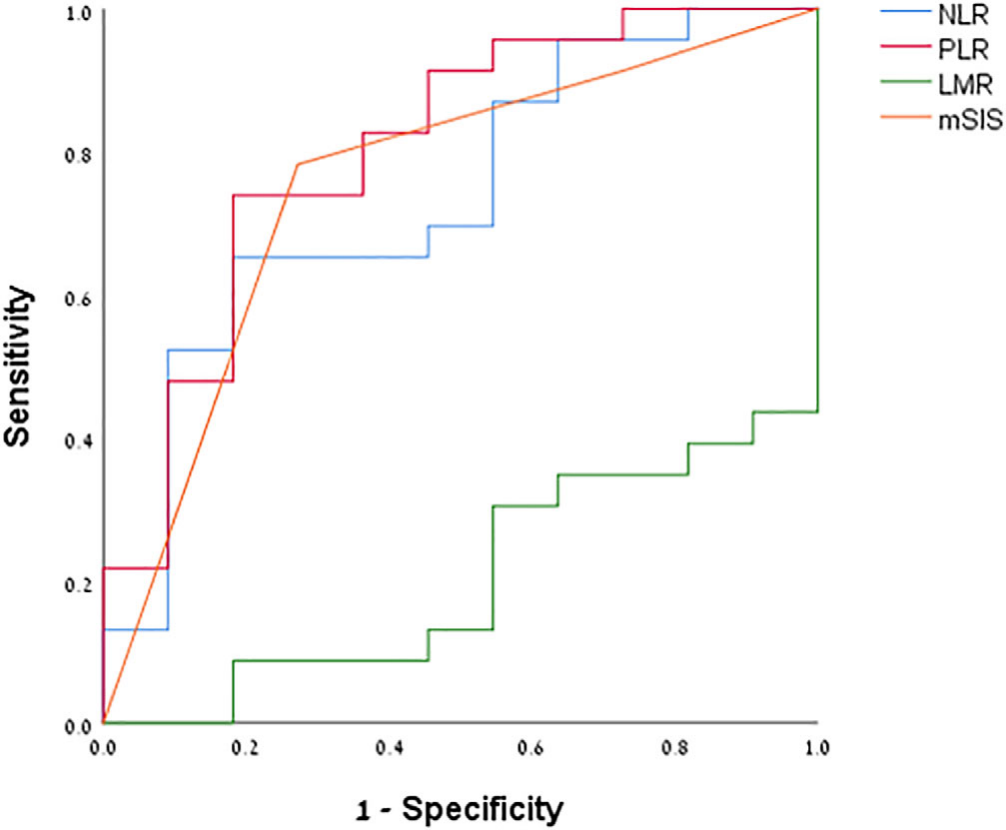
Area under the curve (AUC) analysis of NLR, PLR, LMR and mSIS and disease recurrence in patients who underwent expedited operations. For NLR, *P*‐value = 0.028, Area = 0.735; for PLR, *P*‐value = 0.005, Area = 0.805; for LMR, *P*‐value = 0.005, Area = 0.202; for mSIS *P*‐value = 0.018, Area = 0.753.

Median follow‐up duration was 388 days (range 7–3951 days). Fifty‐six patients (69.1%) had documented recurrence of CD after surgery. AUC analysis demonstrated that among patients who were operated in expedited settings, NLR (*P* = 0.028, *Area* = 0.735), PLR (*p* = 0.005, *Area* = 0.805), LMR (*P* = 0.005, *Area* = 0.202) and mSIS (*P* = 0.018, *Area* = 0.753) were independently associated with disease recurrence (Fig. 2). Cox regression analysis showed that high NLR was an independent prognostic factor for recurrence (*P* = 0.027, *HR* = 1.289, *CI* = 1.029–1.614), but was associated with longer time to recurrence (*P* = 0.024, *HR* = 0.95, *CI* = 0.91–0.99) as presented in Table 5. Age (*P* = 0.826), gender (*P* = 0.7299), smoking status (*P* = 0.11), usage of corticosteroids (*P* = 0.915), TNF‐α inhibitors before operation (*P* = 0.982) and BMI (*P* = 0.07) were not found to be independently associated with recurrence status.

**Table 5:** Cox regression between inflammatory markers and time from surgery to recurrence

	Expedited operation (*N* = 46)		Elective operation (*N* = 35)	
	*P*‐value	Hazard ratio (CI)	*P*‐Value	Hazard ratio (CI)
NLR	0.024*	0.95 (0.91–0.99)	0.304	1.066 (0.94–1.22)
PLR	0.183	0.998 (0.996–1.001)	0.149	1.001 (1–1.003)
LMR	0.822	0.974 (0.78–1.22)	0.152	0.776 (0.55–1.098)
mSIS	0.994	1.002(0.51–1.97)	0.288	1.422(0.74–2.722)

Abbreviations: CI, confidence interval.

* *P* < 0.05.

## Discussion

Surgery is a pivotal part of the management of CD, despite recent advancements in medical therapy.^2^, ^3^ Many patients afflicted by CD undergo surgery under poor nutritional status and active inflammation. Furthermore, many of these patients suffer from long‐standing disease at the time of surgery, and therefore may be malnourished and have impaired wound healing mechanisms.^36^ In addition, although the effects of anti‐inflammatory drugs and biological agents on post‐operative complications remain undetermined, some studies have shown that they may affect healing processes in the body and impair surgical outcomes.^6^ Therefore, patient optimization in CD patients is of high importance.

In this study, we aimed to investigate the clinical and prognostic value of blood cell interaction‐based inflammatory markers that were previously shown to be useful in other benign and malignant conditions in a cohort of CD surgical patients.

We found that higher NLR values were associated with higher incidence of major post‐operative complications and of re‐operation in elective operations, and with higher incidence of thrombo‐embolic events after expedited operations. NLR was found to be associated with disease recurrence but was also associated with longer time to recurrence after surgery, a discrepancy that may be clarified with further analysis in larger cohorts.

In general, NLR values are elevated in states of systemic inflammation. This can be attributed to delayed neutrophil apoptosis due to elevated circulatory antiapoptotic factors,^37^ and increased lymphocyte apoptosis in advanced inflammatory states.^38^ As inflammation and thrombosis are linked, it comes as no surprise that NLR was found to be associated with thrombosis, as demonstrated in previous studies.^39^, ^40^ Prior research has also established a connection between NLR and post‐operative complications in other surgical diseases.^41^ Two previous studies have assessed NLR as a predictor for post‐operative outcomes specifically in CD patients, with contradictory results. Our results concur with the findings of a retrospective research conducted by Kang *et al*., that included 108 CD patients, and showed that in the high NLR group (≥4.1) there was greater incidence of penetrating disease, neutrophilia and lymphopenia, and 2.78 higher risk of post‐operative complications.^29^ Contrarily, in a retrospective research that was conducted by Argeny *et al*. and included 373 CD patients ‐ while higher NLR values were found in patients with penetrating disease and malignancy, patients who developed post‐operative complications surprisingly showed lower NLR values.^30^

We found that PLR was associated with major post‐operative complications and re‐operation rate and correlated with Clavien‐Dindo score in elective patients. PLR is a marker that was initially used in malignant conditions and was also studied in many inflammatory and rheumatic diseases. Thrombocytosis is a hallmark of systemic inflammation, as platelet levels rise in response to inflammatory cytokines such as Interleukin‐6, and in turn release agents that are involved in inflammation and thrombosis.^42^ In contrary, lymphocyte counts are known to be inversely associated with inflammation.^43^ Therefore, higher PLR values represent more severe inflammatory states. PLR was previously shown to be a potential diagnostic tool in CD in a study by Feng *et al*.^28^ and correlated with disease activity as shown in a study by Chen *et al*.^27^ In a retrospective study that was conducted by Lareyre *et al*. and included 244 patients, extreme PLR values were shown to be associated with higher risk of post‐operative complications following abdominal aortic aneurysm repair.^31^ It was not previously tested as a predictor of post‐operative complications in CD.

Lower LMR was associated with higher incidence of thrombo‐embolic events in patients who underwent expedited operations. LMR is a marker that was originally studied in patients with hematologic and solid malignancies. Lymphopenia in these patients represents weak inflammatory cellular response to cancer, that allows for tumour proliferation. Monocytes are known to be regulators of the cancer microenvironment and are increased as the tumour burden grows.^44^ Therefore, higher LMR correlated with better survival in several previous studies. Higher LMR also correlated with better outcomes in benign inflammatory and infectious conditions.^21^, ^22^, ^23^ LMR was not previously studied in CD but was tested as a predictor for post‐operative outcomes in other situations. Our results concur with a study by Zhu *et al*., that showed a correlation between lower LMR values and higher occurrence of post‐operative deep vein thrombosis after total joint arthroplasty.^32^ These findings are compatible with the fact that monocytes are known to link inflammation and procoagulant states in different pro‐thrombotic conditions.^45^

mSIS was associated with higher occurrence of major post‐operative complications in patients who underwent expedited operations. mSIS is a score based on albumin levels, that are known to be elevated in states of systemic inflammation and malnutrition, in combination with LMR. In previous studies, mSIS was shown to have prognostic value in many malignant conditions, and was associated with older age, more comorbidities, larger tumour size, higher TNM stage and higher occurrence of metastases.^24^, ^25^, ^26^ mSIS was not previously studied in benign conditions including CD, or as a predictor for post‐operative complications. Therefore, our results cannot be compared to other studies.

This study has a few limitations, mainly due to the retrospective nature and rather small cohort. However, the findings of this study clearly demonstrate that blood cell interaction based inflammatory markers can play a role in patient evaluation and surgical risk stratification when considering surgical intervention in patients suffering from CD.

In conclusion, we believe that inflammatory markers including NLR, PLR, LMR and mSIS can shed light on the inflammatory status of patients with CD, and aid clinicians in the decision‐making process when treating this complex disease. Larger scale prospective studies are needed to validate and determine the exact role of each specific inflammatory marker. Further studies regarding the association between inflammatory markers and success of pre‐operative optimization process are on their way.

## Conflict of interest

The authors have no conflicts of interest to declare.

## Funding information

No funding was received for conducting this study. The authors have no relevant financial or non‐financial interests to disclose.

## Ethical approval

The study was approved by the local institutional ethics committee (SMC‐1443‐14).

## AUTHOR CONTRIBUTIONS

**Gil Mullin:** Data curation; investigation; project administration; writing – original draft; writing – review and editing. **Roi Anteby:** Writing – review and editing. **Harel Jacoby:** Writing – review and editing. **Ilan Kent:** Writing – review and editing. **Edward Ram:** Writing – review and editing. **Ido Nachmany:** Conceptualization; writing – review and editing. **Nir Horesh:** Conceptualization; supervision; writing – review and editing.
